# Clinical Impact of Unexpected Para-Aortic Lymph Node Metastasis in Surgery for Resectable Pancreatic Cancer

**DOI:** 10.3390/cancers13174454

**Published:** 2021-09-03

**Authors:** Ho-Kyoung Lee, Yoo-Seok Yoon, Ho-Seong Han, Jun Suh Lee, Hee Young Na, Soomin Ahn, Jaewoo Park, Kwangrok Jung, Jae Hyup Jung, Jaihwan Kim, Jin-Hyeok Hwang, Jong-Chan Lee

**Affiliations:** 1 Department of Internal Medicine, Seoul National University Bundang Hospital, Seongnam 13620, Korea; 54408@snubh.org; 2 Department of Surgery, Seoul National University Bundang Hospital, Seongnam 13620, Korea; yoonys@snubh.org (Y.-S.Y.); hanhs@snubh.org (H.-S.H.); rudestock@snubh.org (J.S.L.); 3 Department of Pathology, Seoul National University Bundang Hospital, Seongnam 13620, Korea; 66040@snubh.org; 4 Samsung Medical Center, Department of Pathology and Translational Genomics, Seoul 06351, Korea; soomin17.ahn@samsung.com; 5 Department of Internal Medicine, Seoul National University College of Medicine, Seoul National University Bundang Hospital, Seongnam 13620, Korea; 82410@snubh.org (J.P.); herojkr@snu.ac.kr (K.J.); 82517@snubh.org (J.H.J.); drjaihwan@snubh.org (J.K.); woltoong@snu.ac.kr (J.-H.H.)

**Keywords:** pancreatic cancer, para-aortic lymph node, metastasis

## Abstract

**Simple Summary:**

Para-aortic lymph node (PALN) metastasis in pancreatic cancer (PC) is regarded as a contraindication to surgical resection. Nevertheless, the prognostic impact of unexpected intraoperative PALN metastasis is not firmly established. This retrospective study aims to analyze the prognostic impact of unexpected PALN metastasis and give insight on what surgeons should consider for patients with unexpected intraoperative PALN metastasis.

**Abstract:**

Radiologically identified para-aortic lymph node (PALN) metastasis is contraindicated for pancreatic cancer (PC) surgery. There is no clinical consensus for unexpected intraoperative PALN enlargement. To analyze the prognostic role of unexpected PALN enlargement in resectable PC, we retrospectively reviewed data of 1953 PC patients in a single tertiary center. Patients with unexpected intraoperative PALN enlargement (group A1, negative pathology, *n* = 59; group A2, positive pathology, *n* = 13) showed median overall survival (OS) of 24.6 (95% CI: 15.2–33.2) and 13.0 (95% CI: 4.9–19.7) months, respectively. Patients with radiological PALN metastasis without other metastases (group B, *n* = 91) showed median OS of 8.6 months (95% CI: 7.4–11.6). Compared with group A1, groups A2 and B had hazard ratios (HRs) of 2.79 (95% CI, 1.4–5.7) and 2.67 (95% CI: 1.8–4.0), respectively. Compared with group A2, group B had HR of 0.96 (95% CI: 0.5–1.9). Multivariable analysis also showed positive PALN as a negative prognostic factor (HR 2.57, 95% CI: 1.2–5.3), whereas positive regional lymph node did not (HR 1.32 95% CI: 0.8–2.3). Thus, unexpected malignant PALN has a negative prognostic impact comparable to radiological PALN metastasis. This results suggests prompt pathologic evaluation for unexpected PALN enlargements is needed and on-site modification of surgical strategy would be considered.

## 1. Introduction

Pancreatic ductal adenocarcinoma is the second most common gastrointestinal cancer in the United States and is responsible for 43,000 deaths annually [1]. It is one of the most aggressive tumors, with a 1-year mortality rate of 20–25% [2,3,4]. Approximately 80% of patients with pancreatic cancer (PC) are diagnosed with metastatic lesions. Surgical resection is the only curative treatment for patients with no distant metastasis.

Previous studies on PC have shown that regional lymph node (LN) metastasis results in poor prognosis [5,6,7]. The American Joint Committee on Cancer (AJCC) 8th edition defines N stage according to the number of regional LN metastases [8].

The definition of metastatic LNs depends on the location of the primary tumor, either in the head or tail [8]. In both pancreatic head and pancreatic tail cancers, para-aortic LN (PALN) metastasis is defined as distant metastasis [8]. PALN metastasis may imply systemic illness with aggressive tumor behavior, resulting in a grave prognosis. Previous studies showed that radiologically observed PALN metastasis correlates directly with poor prognosis [9,10,11,12]. Therefore, patients with radiologically observed PALN metastasis preoperatively are recommended to undergo chemotherapy or radiotherapy rather than surgical resection.

Nevertheless, when surgeons discover unexpected PALN metastasis during surgery, which was not recognized in preoperative imaging studies, and there is no definite consensus on whether there should be any change in the treatment strategy. In this study, we evaluated the prognostic value of unexpected PALN metastasis in patients with clinically resectable PC with no other distant metastases.

## 2. Materials and Methods

### 2.1. Patients

Medical and pathologic records of patients diagnosed with pancreatic adenocarcinoma in a single tertiary center (Seoul National University Bundang Hospital) from 2004 to 2019 were retrospectively reviewed. Patients who were previously diagnosed with PC and treated with chemotherapy, radiotherapy, or surgery from other hospitals were excluded from the study. Patients with malignant tumors other than PC were also excluded.

Results of all imaging tests were reviewed and the patients categorized in accordance with the AJCC 8th edition [8]. Patients diagnosed with locally advanced PC and borderline resectable PC were excluded. Patients with metastatic PC were divided into those with PALN metastasis alone and those with other distant metastases.

Surgical records and pathology reports of patients with radiologically resectable PC, with clinical stage TxN0M0, were reviewed. In the surgery, all observed PALN enlargement were evaluated by their gross morphology. PALN satisfying all four following conditions were considered benign or reactive: (1) smaller than 10 mm in diameter, (2) pinkish in color, (3) soft or tender on palpation, (4) consistent size and morphology to adjacent lymph nodes. All other PALN not meeting all four conditions were harvested, and sent for pathologic evaluation.

**Figure 1:**
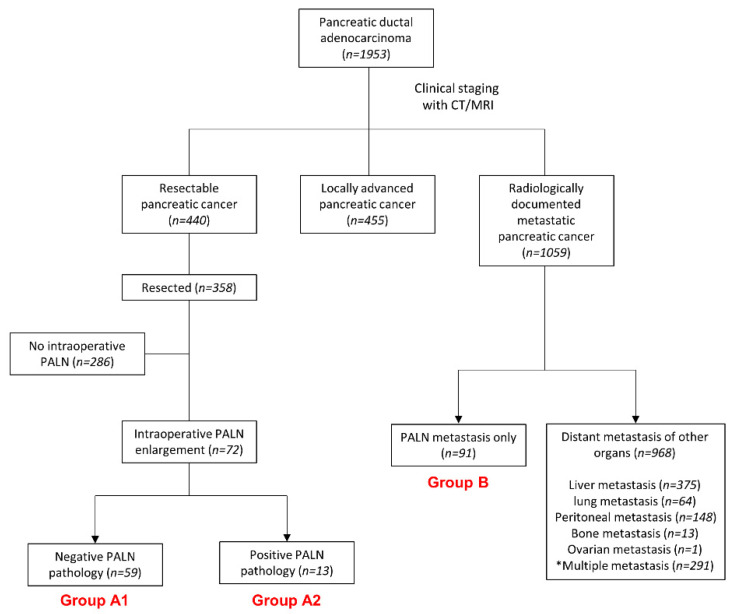
Flowchart of the patient selection process. * This group included patients with PALN metastasis and other metastases (PALN: para-aortic lymph node).

Among patients who underwent surgical resection, those who had unexpected intraoperative PALN enlargement during surgery were categorized as group A. All patients in group A had para-aortic LN dissection, and the final pathologic diagnosis was made. Patients with negative para-aortic LNs were categorized as group A1, while those with positive para-aortic LNs were categorized as group A2 (Figure 1). Patients with no other distant metastasis, but only PALN metastasis, were classified as patient group B.

### 2.2. Study Design

This study was conducted as a single-center, retrospective cohort study. The primary endpoint was the overall survival of patients.

### 2.3. Statistical Analysis

Statistical analyses were performed using Stata version 15.0. Categorical data on the three patient groups were analyzed using a 2 × 3 chi-square test or 2 × 3 Fisher’s exact test. Numerical data with a normal distribution were analyzed using analysis of variance, and those that failed to follow a normal distribution were analyzed using the Kruskal–Wallis test. Survival data were analyzed using the Cox proportional hazard model. Categorical variables are expressed as percentages, and continuous variables are expressed as IQR. A *p*-value < 0.05 was considered to indicate statistical significance.

## 3. Results

### 3.1. Baseline Characteristics

A total of 1953 patients were diagnosed with pancreatic ductal adenocarcinoma from January 2004 to December 2019 (Figure 1). Patients were categorized according to their clinical stages. In the clinical staging based on the imaging studies, four hundred-forty patients were diagnosed with resectable PC, 455 were diagnosed with locally advanced PC, and 1059 were diagnosed with metastatic PC.

Among 440 resectable PC patients, a total of 358 patients underwent curative resection for pancreatic cancer. Among them, unexpected intraoperative PALN enlargement was found in 72 patients, who were categorized as group A. Based on the final pathologic report, patients with benign PALN enlargements were categorized into group A1 (*n* = 59), and those with malignant PALN were categorized into group A2 (*n* = 13). Among 1059 patients with metastatic PC, ninety-one patients had no distant metastasis other than PALN metastasis and were categorized as group B.

No significant difference was observed in the baseline demographic information among the three patient groups (Table 1).

### 3.2. Survival of Patients with Unexpected PALN Enlargements

**Figure 2:**
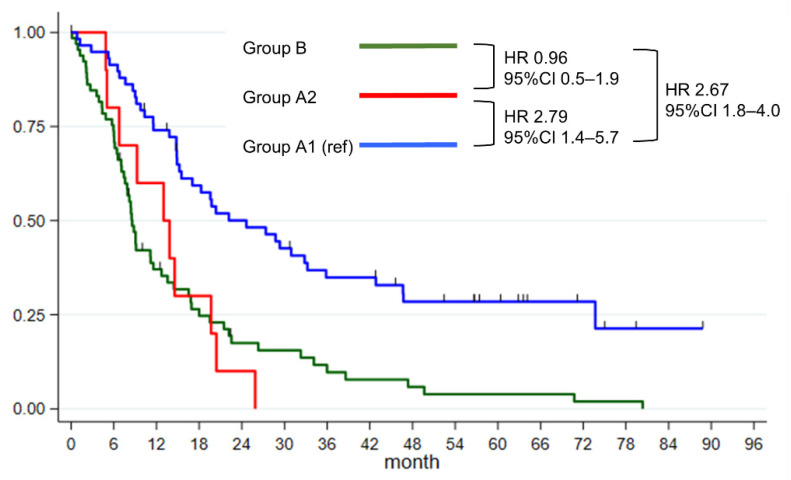
Kaplan–Meier survival analysis of the three patient groups. Group A1, unexpected PALN with negative pathology; Group A2, unexpected PALN with positive pathology; Group B, metastatic PALN in imaging study.

Figure 2 and Table 2 shows the overall survival of the three patient groups. The median survival of patients in group A1 was 24.6 months, and that of patients in group B was 8.6 months (hazard ratio [HR]: 2.67, 95% confidence interval [CI]: 1.8–4.0, *p* < 0.001 compared to group A1). On the contrary, the median survival of patients in group A2 was 13.0 months (HR: 1.04, 95% CI: 0.7–1.4, *p* = 0.905, compared to group B). Overall survival of patients in group A2 were shorter than patients in group A1 (HR: 2.79, 95% CI: 1.4–5.7, *p* = 0.005, compared to group A1).

### 3.3. Other Prognostic Factors Affecting Overall Survival

Table 2 shows other factors affecting the overall survival. Sex, body mass index (BMI) at diagnosis, initial carcinoembryonic antigen (CEA), and tumor size failed to show a significant difference in patient survival. No significant survival difference was observed between patients in group B and those in group A2 (HR: 0.96, 95% CI: 0.5–1.9, *p* = 0.905). Patients in group B had significantly worse prognosis than those in group A1 (HR: 2.67, 95% CI: 1.8–4.0, *p* < 0.001).

On multivariable analysis, initial CA19-9 level or regional LN status failed to show prognostic significance. Multivariable analysis still showed shorter overall survival in group A2 compared to that in group A1 (HR: 2.57, 95% CI: 1.2–5.3, *p* = 0.010).

### 3.4. Surgical Information including Postoperative Complication

Surgical information of patients in groups A1 and A2 is summarized in Table 3. Patients in group B did not go through curative surgery, and has no pathologic data on PALN. No significant differences were found in the operation type, the most common being pancreaticoduodenectomy. The total numbers of LNs resected were 24 in group A1 and 25 in group A2. The numbers of para-aortic LNs resected were 6 in group A1 and 3 in group A2.

The positive LN ratio (LNR) is defined as the ratio of the number of positive LNs to the total number of LNs harvested during surgery. The median LNR in group A1 was 5.6% and that in group A2 was 26.7% (*p* = 0.0002).

The number of surgical complications did not differ between the two patient groups (Table 3). The most common surgical complications in the two patient groups were pancreatic fistula, followed by surgical site infection and postoperative hemorrhage.

### 3.5. Effects of Overall Lymph Node Status

**Figure 3:**
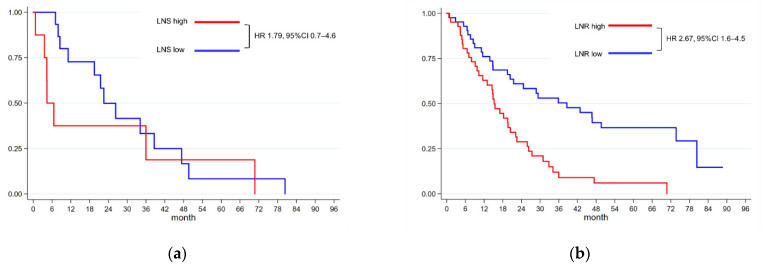
Kaplan–Meier survival analysis according to the status of lymph node metastasis. (**a**) Survival analysis for patients with ≤3 lymph node metastasis and patients with ≥4 lymph node metastasis. LNS low: median survival 22.5 months; LNS high: median survival 4.4 months; HR: 1.79, 95% CI: 0.7–4.6, *p* = 0.233. (**b**) Survival analysis of patients with LNR of <4.42 and patients with LNR of ≥4.42. LNR low (≤4.42): median survival 38.6 months; LNR high (>4.42): median survival 15.2 months; HR: 2.67, 95% CI: 1.6–4.5, *p* < 0.001.

Patient survival was analyzed based on the number of metastatic LNs (LNS) (Figure 3A). Patients were grouped as follows: those with fewer than four metastatic LNs and those with more than three metastatic LNs. The median survival of patients with less than four metastatic LNs was 22.5 months, and that of patients with more than three metastatic LNs was 4.4 months (HR: 1.79, 95% CI: 0.7–4.6, *p* = 0.233).

Patient survival was also analyzed based on the LNR (Figure 3B). The median LNR was 4.2%. We categorized the patients into two groups: patients with LNR ≤ 4.2% and patients with LNR > 4.2%. The median survival of patients with lower LNR was 38.6 months and that of patients with higher LNR was 15.2 months (HR: 2.67, 95% CI: 1.6–4.5, *p* < 0.001).

### 3.6. Other Medical Information

Non-surgical therapies performed in patients are summarized in Table 4. The most common first-line palliative chemotherapy regimen was FOLFIRINOX. Gemcitabine monotherapy was the most common adjuvant therapy regimen.

The ratio of patients receiving radiotherapy varied among the three patient groups. Four out of 91 patients in group B (4.4%) received radiotherapy, 1 out of 13 patients in group A2 (7.7%), while 19 out of 59 patients in group A1 (32.2%) received radiotherapy.

## 4. Discussion

This study was a retrospective analysis of the prognostic role of unexpected intraoperative PALN metastasis in PC. In our study, unexpected intraoperative PALN metastasis resulted in shorter patient survival. On multivariable analysis, the presence of regional lymph node metastasis did not significantly affect overall patient survival.

In PC, the prognostic role of distant metastasis has been well established in various studies [13,14,15]. Nevertheless, the prognostic value of PALN metastasis has not been firmly established [9,10,11,16,17]. When unexpected PALN enlargement is found during curative resection of PC, there is no consensus on whether additional treatment strategies will be implemented. A systematic review and meta-analysis by Paiella suggested that PALN metastasis correlates with poor prognosis in patients with pancreatic adenocarcinoma [9]. On the contrary, a multicenter study by Masayuki Sho suggested that some patients with PC having metastatic PALN may survive longer than expected after undergoing pancreatectomy [10,16]. Moreover, the prognosis of patients with ‘PALN metastasis only’ without other metastases remains unclear.

In this study, the patients were primarily categorized into two groups: group A, patients with radiologically resectable PC who had unexpected intraoperative PALN enlargement; and group B, patients with clinical metastatic PC having only PALN metastasis, with no evidence of other distant metastases. Patients in group A were further grouped into group A1, comprising patients with pathologically benign PALN, and group A2, comprising patients with pathologically proven PALN metastasis.

The median survival of patients in group B (median: 8.6 months, 95% CI: 15.2–33.2 months) was similar to historical data of that of metastatic PCs [14,15,18,19]. Group A1 also showed similar overall survival of historical data of resectable PC [18,19]. Therefore, median survival of patients in group B were significantly shorter than that of patients in group A1 (HR: 2.67, 95% CI: 1.8–4.0, *p* < 0.001). The survival of patients in group B were not different from those of patients in group A2 (HR: 0.96, 95% CI: 1.1–2.5, *p* = 0.905).

Patients in group A2 had significantly shorter median survival compared to that of group A1 (HR: 2.79, 95% CI: 1.4–5.7, *p* = 0.005). This result suggests negative prognostic impact of unexpected PALN metastasis. Surgeons are recommended to perform frozen biopsy for unexpected intraoperative PALN enlargements. This result is consistent with that of a systemic review by Paiella [9].

A retrospective study by Hyoung Woo Kim et al. studied whether early adjuvant treatment after surgical resection yields better outcomes in patients with PC [20]. The patients with early adjuvant treatment had significantly prolonged overall survival and disease-free survival. They suggest that adjuvant treatment be delivered earlier and completed for better outcomes in PC patients. We have shown that intraoperative PALN metastasis has worse prognostic impact comparable to radiologically observed PALN metastasis. Taking both studies into consideration, when unexpected intraoperative PALN metastasis are found, surgeons should consider the risk of surgical complication, which might delay following adjuvant treatment. Further studies are needed to clarify whether changing to palliative surgery would result in fewer surgical complication, earlier adjuvant treatment, and thereby results in improved patient prognosis. We have proposed in supplementary figure a treatment algorithm according to the status of para-aortic lymph node in pancreatic cancer (Figure S1).

In addition to PALN metastasis, the number of regional LN metastases is an important confounding factor in this study. Various studies have reported that lymph node ratio, rather than absolute number of metastatic LN is inversely associated to survival [21,22,23]. No significant survival differences were found according to the number of metastatic LNs. Our data also showed that there were no significant survival differences according to the absolute number of positive LNs. On the contrary, patients with lower LNR had better prognosis than those with higher LNR.

There has been previous studies regarding ‘optimal extent’ of lymphadenectomy on patients with resectable PC because the extent of lymphadenectomy need to be balanced with the risk of surgical complication [24]. Warschkow et al. reported in an observational study that ‘extensive lymphadenectomy itself’ improves patient survival, in both patients with or without metastatic regional lymph node [25]. Combining the results of previous studies and those of the present study, it is suggested that surgeons harvest a sufficient number of LNs during surgery.

In terms of adjuvant chemotherapy, the proportion of patients receiving adjuvant chemotherapy was 5/13 (38.5%) in group A2, which was lower than 35/59 (59.3%) on group A1 (*p* = 0.012). This difference might come from the post-operative deterioration of patients’ performance, which make it difficult for patients to receive adjuvant chemotherapy. Among patient groups, some received 5-FU based regimen and others received gemcitabine-based regimens. This would be because we enrolled the patients between 2004 to 2019, which includes both gemcitabine-era and beyond.

This study has a few limitations. The number of patients was relatively small. Nevertheless, the statistical analysis resulted in significant survival differences, and further studies with larger numbers of patients are warranted to yield more significant differences. Not all patients in the study had information on frozen biopsy, and we analyzed the PALN metastasis status based on the final pathologic report. Nevertheless, Alexandre Doussot reported that frozen sections of PALN yielded accurate PALN assessment [26,27,28,29].

## 5. Conclusions

Unexpected malignant PALN could have a negative prognostic impact on survival of patients with radiologically resectable PC comparing to those with clinically metastatic PALN. This study suggested that frozen sections need to be performed when unexpected PALN enlargement is found during surgery. When patients have unexpected intraoperative PALN metastasis, surgeons could adopt two options of on-site strategy including scaling down surgery for early initiation of palliative chemotherapy, as well as planned curative resection with adjuvant chemotherapy.

## Figures and Tables

**Table 1:** Patients’ baseline characteristics.

Variable	Group A1 (*n* = 59)	Group A2 (*n* = 13)	Group B (*n* = 91)	Total Patients (*n* = 163)	*p*-Value
Age (years)	63 (53–72)	60 (57–66)	67 (60–76)	65 (58–74)	0.321
Sex					0.790
Male	29 (49.1)	5 (38.5)	45 (49.5)	79 (48.5)	
Female	30 (50.8)	8 (61.5)	46 (50.6)	84 (51.5)	
BMI	22.1 (20.5–23.9)	21.30 (19.7–23.5)	22.58 (20.7–25.0)	22.33 (20.5–24.8)	0.331
Initial tumor markers					
CA 19-9	101.8 (38.2–430)	191.9 (41–1653.75)	240 (74–731.3)	180.9 (49–607.5)	0.081
CEA	2.9 (1.4–5.15)	4.9 (3.6–5.4)	2.55 (1.9– 5)	2.8 (1.65–5.1)	0.302
Tumor location					0.175
Head	49 (83.1)	13 (100)	62 (68.1)	124 (76.1)	
Body	7 (11.9)	0	15 (16.5)	22 (13.5)	
Tail	2 (3.3)	0	11 (12.1)	13 (8.0)	
Multiple	1 (1.7)	0	3 (3.3)	4 (2.4)	
Tumor size (cm)	2.7 (2–3.3)	2.7 (2.1–3.3)	3.2 (2.5–4.2)	3.00 (2.4–3.9)	0.102
Concomitant regional lymph node *	5 (8.5)	1 (7.7)	39 (42.9)	45 (27.6)	<0.001

Data are presented as median (interquartile range) or number of patients (%), unless otherwise stated. * Concomitant regional lymph node refers to radiologically observed regional LN enlargement. Post hoc analysis showed no difference between group A1 and A2 (*p*-value = 0.891).

**Table 2:** Univariable and multivariable analyses.

Variable (*n*)	Median Survival (month)	95% CI	Univariable			Multivariable		
			HR	95% CI	*p*-Value	HR	95% CI	*p*-Value
Overall patients	14.6	11.1–17.1						
Sex								
Male (67)	16.9	11.5–20.4	–					
Female (67)	13.0	8.8–15.5	0.92	0.6–1.3	0.680			
Age								
Age ≤ 65 (65)	19.6	14.9–27.4	–			–		
Age > 65 (69)	8.7	6.6–13.0	1.98	1.4–2.9	<0.001	1.73	1.1–2.7	0.012
BMI								
BMI ≤ 22.33 (61)	14.8	10.3–17.1	–					
BMI > 22.33 (54)	21.5	11.1–29.3	0.70	0.5–1.1	0.089			
Group (ref A1)						–		
Group A1	24.6	15.2–33.2	–			–		
Group A2	13.0	4.9–19.7	2.79	1.4–5.7	0.005	2.57	1.2–5.3	0.010
Group B	8.6	7.4–11.6	2.67	1.8–4.0	<0.001	1.72	1.0–2.9	0.041
Group (ref A2) *								
Group B	24.6	15.2–33.2	0.96	0.5–1.9	0.905			
Regional lymph node								
No metastasis	14.9	11.2–20.4						
Metastasis	11.6	7.9–16.5	1.69	1.1–2.5	0.010	1.32	0.8–2.3	0.315
CA19–9								
CA19–9 ≤ 180 (60)	21.5	14.5–28.8	–			–		
CA19–9 > 180 (56)	11.6	9.2–15.5	1.70	1.1–2.6	0.011	1.49	0.97–2.3	0.067
CEA								
CEA ≤ 2.8 (39)	15.5	11.2–21.5	–					
CEA > 2.8 (51)	13.0	8.0–18.3	1.16	0.7–1.8	0.543			
Tumor size								
Tumor size ≤ 3.0 (59)	14.8	11.2–24.6						
Tumor size > 3.0 (75)	13.0	8.5–18.0	1.43	1.0–2.1	0.069			

* Not applied in the multivariable analysis.

**Table 3:** Surgical information of groups A1 and A2.

Patient Group (*n*, %)	A1 (59, 100%)	A2 (13, 100%)	*p*-Value
OP type			0.477
Pancreaticoduodenectomy *	47 (80%)	11 (85%)	
Distal pancreatectomy	5 (8.5%)	0 (0%)	
Total pancreatectomy	5 (8.5%)	1 (7.5%)	
Others ^†^	2 (3.4%)	1 (7.5%)	
Resection margin			0.272
Negative	41 (69%)	6 (46%)	
Positive	14 (24%)	5 (38%)	
Overall number of dissected LN (median, IQR)	24 (16–33)	25 (15–32)	0.915
No of harvested PALN	6 (2–9)	3 (1–5)	0.117
No of harvested regional LN	18 (11–25)	22 (12–31)	0.476
Pathologic T staging			0.505
Tx	0 (0%)	2 (15%)	
T1–T2	47 (80%)	10 (77%)	
T3–T4	12 (20%)	1 (8%)	
Pathologic N staging			0.191
Nx	2 (3%)	2 (15%)	
N0	27 (46%)	0 (0%)	
N1	33 (56%)	3 (23%)	
N2	7 (12%)	8 (62%)	
Ratio of positive regional LN (%)			
	8.1 (0–30.7)	26.67 (0–51.9)	0.0002
Pathologic M staging ^‡^			
M0	58 (98%)	0 (0%)	
M1	1 (2%)	11 (100%)	
Moderate to severe surgical complication			
All complication	32 (54%)	4 (31%)	0.163
Surgical site infection	4 (7%)	1 (8%)	0.826
Postoperative hemorrhage	4 (7%)	0 (0%)	0.831
Pancreas fistula	9 (15%)	0 (0%)	0.344
Liver abscess	1 (2%)	0 (0%)	0.647
Others ^§^	14 (24%)	3 (23%)	

Data are presented number of patients (%), unless otherwise stated. * Pancreaticoduodenectomy includes PPPD, PRPD, and Whipple operation. ^†^ Others included palliative cholecystectomy and O&C. ^‡^ As groups A1 and A2 had no metastasis other than distant LN metastasis, M stage depends only on the presence or absence of distant LN metastasis. ^§^ Others include chylous ascites, bacteraemia, acute kidney injury, postoperative ileus, and cholangitis. (PALN: para-aortic lymph node).

**Table 4:** Treatment information other than surgery.

Patient Group (*n*, %)	A1 (59)	A2 (13)	B (91)	*p*-Value
First-line palliative chemotherapy				0.918
FOLFIRINOX	2 (3.4%)	2 (15.4%)	25 (27.5%)	
Gemcitabine with nab-paclitaxel	0 (0%)	0 (0%)	5 (5.5%)	
Gemcitabine monotherapy	1 (1.7%)	1 (7.7%)	6 (6.6%)	
Other gemcitabine-based chemotherapy	3 (5.1%)	1 (7.7%)	12 (13.2%)	
Adjuvant chemotherapy *				0.012
Modified FOLFIRINOX	1 (1.7%)	1 (7.7%)	3 (3.3%)	
Other 5-FU-based regimen	1 (1.7%)	0 (0%)	0 (0%)	
Gemcitabine monotherapy	28 (47.5%)	3 (23.1%)	6 (6.6%)	
Other gemcitabine-based chemotherapy	5 (8.5%)	1 (7.7%)	3 (3.3%)	
Pre-operative chemotherapy				0.230
FOLFIRINOX	2 (3.4%)	1 (7.7%)	0 (0%)	
Gemcitabine monotherapy	0 (0%)	0 (0%)	0 (0%)	
Gemcitabine + Erlotinib	2 (3.4%)	0 (0%)	0 (0%)	
Radiation therapy				<0.001
Yes	19 (32.2%)	1 (7.7%)	4 (4.4%)	
No	40 (67.8%)	12 (92.3%)	87 (95.6%)	

Data are presented as number of patients (%). * Palliative chemotherapy after tumor recurrence is excluded.

## Data Availability

The data presented in this study are available on request from the corresponding author. The data are not publicly available due to privacy issues.

## Supplementary Materials

The following are available online at https://www.mdpi.com/article/10.3390/cancers13174454/s1, Figure S1: Treatment algorhithm in accordance to para-aortic lymph node status. (PC: pancreatic cancer; PALN: para-aortic lymph node).
