# Supplementary material for: Clinical Impact of Unexpected Para-Aortic Lymph Node Metastasis in Surgery for Resectable Pancreatic Cancer
Source: Cancers (Basel). 2021 Sep 3;13(17):4454. doi: 10.3390/cancers13174454 (PMC8431119; doi:10.3390/cancers13174454)
Supplement: Supplementary file 1 [file cancers-13-04454-s001.zip › cancers-1346046-supplementary.pdf]

cancers

Supplementary Material: Clinical Impact of Unexpected Para-Aortic Lymph Node Metastasis in Surgery for Resectable Pancreatic Cancer

Ho-Kyoung Lee, Yoo-Seok Yoon, Ho-Seong Han, Jun Suh Lee, Hee Young Na, Soomin Ahn, Jaewoo Park, Kwangrok Jung, Jae Hyup Jung, Jaihwan Kim, Jin-Hyeok Hwang and Jong-Chan Lee

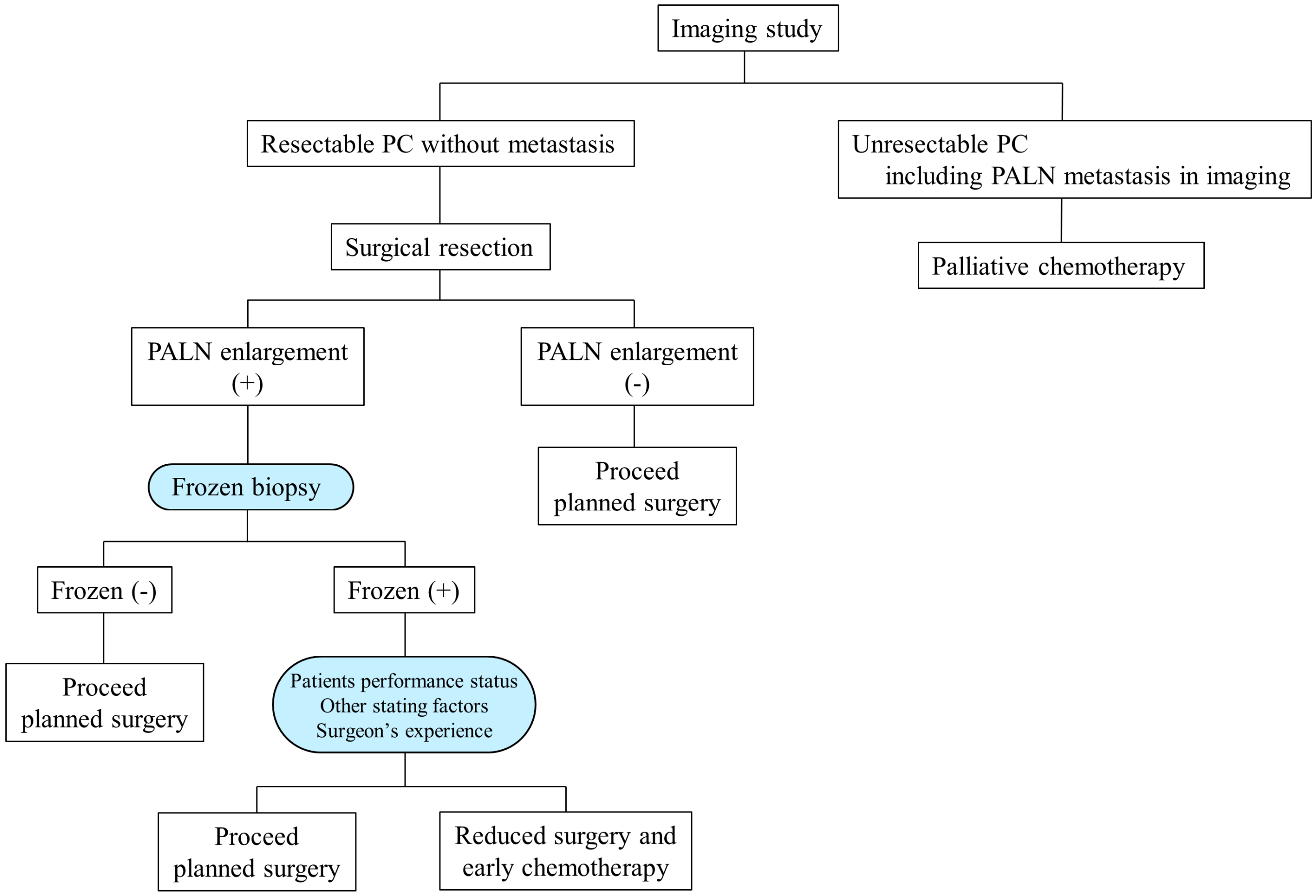

Figure S1. Treatment algorhithm in accordance to para-aortic lymph node status. (PALN: para-aortic lymph node).
